# Suggestions for a Systematic Regulatory Approach to Ocean Plastics

**DOI:** 10.3390/foods10092197

**Published:** 2021-09-16

**Authors:** Margherita Paola Poto, Edel Oddny Elvevoll, Monica Alterskjær Sundset, Karl-Erik Eilertsen, Mathilde Morel, Ida-Johanne Jensen

**Affiliations:** 1 Faculty of Law, UiT—The Arctic University of Norway, N-9037 Tromsø, Norway; mathilde.d.morel@uit.no; 2 The Norwegian College of Fishery Science, Faculty of Biosciences, Fisheries and Economics, UiT—The Arctic University of Norway, N-9037 Tromsø, Norway; edel.elvevoll@uit.no (E.O.E.); karl-erik.eilertsen@uit.no (K.-E.E.); idaj.jensen@ntnu.no (I.-J.J.); 3 Department of Arctic and Marine Biology, UiT—The Arctic University of Norway, N-9037 Tromsø, Norway; monica.a.sundset@uit.no; 4 Department of Biotechnology and Food Science, Norwegian University of Science and Technology, NTNU, N-7491 Trondheim, Norway

**Keywords:** Plasticene, seas, science, regulation, sustainability, multi-tiered, system, food, health

## Abstract

The research investigates the problems and maps the solutions to the serious threat that plastics pose to the oceans, food safety, and human health, with more than eight million tons of plastic debris dumped in the sea every year. The aim of this study is to explore how to better improve the regulatory process of ocean plastics by integrating scientific results, regulatory strategies and action plans so as to limit the impact of plastics at sea. Adopting a problem-solving approach and identifying four areas of intervention enable the establishment of a regulatory framework from a multi-actor, multi-issue, and multi-level perspective. The research methodology consists of a two-pronged approach: 1. An analysis of the state-of-the-art definition of plastics, micro-, and nanoplastics (respectively, MPs and NPs), and 2. The identification and discussion of loopholes in the current regulation, suggesting key actions to be taken at a global, regional and national level. In particular, the study proposes a systemic integration of scientific and regulatory advancements towards the construction of an interconnected multi-tiered (MT) plastic governance framework. The milestones reached by the project SECURE at UiT - The Arctic University of Norway provide evidence of the strength of the theory of integration and rights-based approaches. The suggested model holds substantial significance for the fields of environmental protection, food security, food safety, and human health. This proposed MT plastic governance framework allows for the holistic and effective organization of complex information and scenarios concerning plastics regulation. Containing a clear definition of plastics, grounded on the precautionary principle, the MT plastic framework should provide detailed mitigation measures, with a clear indication of rights and duties, and in coordination with an effective reparatory justice system.

## 1. Introduction

This study takes place within the broad context of the ‘Plastics Age’, also known as the ‘Plasticene’, characterized by an exponential increase of plastic deposits on the planet since the mid-1940s [1,2]. Scientists have been investigating the effects of the escalation of plastic pollution on the planet [2,3,4], and the impacts of plastic on the marine environment. Currently, there is at least eight million tons of plastic dumped in the ocean every year (IUCN 2021 [5]). There is consensus among the preeminent studies on the topic for the need for further research to explore the development of an effective and coordinated regulatory strategy to efficiently reduce plastic pollution on seas and lands [6,7,8,9,10,11].

A prerequisite and *condicio sine qua non* of the regulation process, in the absence of scientific certainty of harmful impacts of plastics at sea, is the reference to the precautionary principle and the need to take preventive urgent action. The precautionary principle is enshrined in Article 15 of the Rio Declaration on Environment and Development [12] and Article 191(2) of the Treaty on the Functioning of the EU [13]. The need for urgent action is especially evident in the case of the unknown impacts of the smallest particles of plastics: microplastics (MPs, less than 5 mm in size) and nanoplastics (NPs with a size defined as either less than 100 or 1000 nm) [14]. Due to the dearth of coordination between the research on the harmful impacts, one may argue that there is not yet sufficient evidence to substantiate a regulatory effort that tackles all the risks that plastics pose to food security, food safety, and ultimately to human health [14]. Consequently, the precautionary principle and the need to take preventive measures can legitimize the policymakers’ anticipatory action even under scientific uncertainty.

Based on this premise, the plastic regulatory effort needs to be methodologically sound and constructed under the multilevel environmental governance (MEG) system [15,16]. MEG combines top-down and bottom-up approaches to achieve an effective reduction of some forms of plastics at the global, regional, and local levels [15]. In this framework, we argue that plastics governance can be developed in at least in three dimensions: through *multi-level*, *multi-actor*, and *multi-issues* perspectives. For clarity, hereinafter these three dimensions or perspectives are grouped under the broad category of ‘multi-tiered (MT) plastics governance’. An MT enforcement system provides criminal, civil, and administrative sanctions in case of non-compliance on the one side (hence the reference to a *multi-issues* dimension). On the other side, it brings together a diverse group of responsible and impacted actors (here the *multi-actor* and *multi-level* dimensions), in the environmental justice movement [17]. The reasoning behind the latter approach stems from the conclusions reached by a UNEP Report that demonstrates how micro and nanoplastic pollution is now in the public domain as an issue of global concern [18]. Consequently, the global fight against plastic pollution, much like the environmental justice movement, is characterized by a multi-actor-driven process [18]. A pluri-subjective regulatory system entails the need for coordinated actions, for provisions on civil society suit authority, collective actions, and promotion of youth campaigns against global inaction on plastics waste [19].

In addition, the provisions of such a complex framework are to be coordinated with the pre-existing legislation on marine environmental protection, food safety, and, in general, conceptualized through the lens of the Agenda 2030 and its sustainable development goals (SDGs): in particular, but not exclusively, SDG 2 (Zero Hunger), 3 (Health), 12 (Sustainable Consumption and Production) and 14 (Life Below Water) [20].

For all the above-mentioned reasons, the present study intends to address the needs highlighted by the preeminent literature. It does so by prospecting the need to develop a regulatory model for the reduction and the sustainable management of marine plastic pollution. The model integrates the “multitude of multi-tiered approaches” (da Costa et al. [11]) thus preventing some of the impending long-term impacts that pollution of the marine environment can have on food security, nutrition, and health.

## 2. Materials and Methods

### 2.1. Problem-Solving Approach

It is possible to identify at least four issues and four corresponding approaches in the problem-solving process related to plastic regulation. Two issues are related to the previously mentioned difficulty of integrated approaches to plastics from hard sciences and law: first, scientific research on the impacts of plastics on seafood security, food safety, and human health is still in its infancy (1). Second, this circumstance hinders regulatory efforts causing a substantive divide between scientific research and regulatory interventions (2). There are two additional issues resulting from endogenous legal factors: plastic regulation has not yet been fully framed and consistently constructed at the global, regional, and local level, which results in fragmented legislation and consequently uncoordinated enforcement (3). Moreover, plastic regulation is generally characterized by a weak level of effectiveness both in terms of time and enforceability of the intervention measures (4) [14,15].

Such highlighted issues can be solved through a regulatory initiative that is grounded on the precautionary principle and that develops along the lines of a systematic and rights-based approach. In particular, the precautionary principle and preventive measures can help overcome issues (1) and (2) and support a framework that overcomes the divide between science and law. A way out of fragmentation and scarce enforceability (respectively, issues (3) and (4)) can be achieved in the systems thinking approach in conjunction with the rights-based approach.

The research design involves the following steps: a. Drawing insights from the definition of plastics and the impacts on the marine environment, nutrition, and health (Section 2.1); b. Understanding the state-of-the-art of plastic regulation (Section 2.2); and c. Combining the two understandings with the proposal of an integrated framework based on systematic approaches (Section 2.3). In particular this third step builds upon several aspects of the preliminary results from the project SECURE at UiT—The Arctic University of Norway on Novel Marine Resources for Food Security and Food Safety [21].

### 2.2. Drawing Insights from the Definition of Plastics and Its Impacts on the (Marine) Environment, Food Security, Food Safety, and Human Health

#### 2.2.1. Definition

Steps towards the creation of a working definition of plastics that could be enshrined in legislation have been facilitated by the efforts of Nanna Hartmann et al. [10]. The research group has established seven criteria for the identification of plastics that threaten the marine environment at large, criteria that must be considered in the assessment of plastic materials: 1. Chemical composition; 2. Solid-state; 3. Solubility; 4. Size; 5. Shape and structure; 6. Color; and 7. Origin [10]. In the view of the researchers, the combination of these criteria and their specifications should facilitate the definition of what plastic is (an inert synthetic polymer) and of its potential harms to the environment [10]. Further research has been conducted by Mitrano et al. (2020) towards the construction of an epistemological basis for regulation, specifically focusing on the impacts of plastic on the marine food web [9]. According to the study, microplastics (MPs) are debris with sizes below 5 mm, resulting from the release of small particles and the fragmentation of larger plastics (respectively, primary and secondary MPs [9]). As for the definition of nanoplastics (NPs), experts refer to the European Food Safety Authority (EFSA)’s opinion of 2016 [22] in which the term is associated with any plastic debris with size between 1 and 100 nm, produced by the degradation of MPs or released directly from domestic and industrial sources [23]. The research highlights the importance of adopting a regulatory approach on the onset of MPs and NPs definitions and throughout the development of a grounded theory for a coordinated framework of the impacts of these on the environment, food security, food safety and human health.

#### 2.2.2. Impacts

Petroleum-based polymer materials, such as polypropylene, polyester, polyethylene and polystyrene have been used as plastics packaging for many years, replacing other packaging materials and increasing by 20-fold since the mid-sixties [24,25]. The scientific community has not reached a consensus on the full extent of threats that plastic—and especially MPs and NPs—pose to the (marine) environment, the food web, and ultimately human health [26,27]. Hence, very little is known about how acute and chronic exposure to MPs and NPs (through, e.g., seafood or plastic bottle drinks) will affect global health [26]. Humans are estimated to ingest 52,000 MPs per year and inhale 69,000 MPs per year through plastic-contaminated air [28]. Most of the ingested MPs and NPs are expected to pass through the digestive tract, and only smaller NPs may potentially enter the blood stream circulation. The impact of a continual presence of MPs and NPs in the human gut is unknown, but to date the research indicates that very high intakes can cause inflammation of the gut lining [29]. Furthermore, organic pollutants can also be absorbed onto MPs and NPs and may enhance the effective uptake and toxicity of these pollutants. Similarly, metallic toxins (cadmium and mercury) and toxic trace elements may also interact with plastic particles serving as vectors for toxic uptake by living organisms [29].

Bio-based plastic packaging materials (natural biopolymers extracted from plants, animals, or microorganisms, including cellulose, starch, milk proteins, polysaccharide gums), can reduce climate impact. However, they may also have less favorable effects when it comes to other environmental impacts such as eutrophication (the gradual increasing of plant nutrients in an aging aquatic ecosystem), use of water, pesticides, and effects on biodiversity [25].

The described set of impacts is heterogeneous and fragmented, resulting in a lack of coordination between scientific research and regulatory intervention [3,4,5,6,7]. At the root of the inconsistencies between measured data and consequent actions is the lack of a common terminology that supports scientific evidence on such threats [7,8]. Moreover, reviews from the EFSA [22], the Food and Agriculture Organization (FAO) [30], the Science Advice for Policy by European Academies (SAPEA) [31], and the Norwegian Scientific Committee for Food and Environment (VKM) [32], provide little knowledge about the presence of NPs in the marine water and seafood, since the methods for either their detection or quantification have not been established yet [22,33]. NPs are expected to be absorbed in the gastrointestinal tract and may end up damaging different organs in marine organisms [30]. Thus, NPs are considered the most dangerous plastics possibly posing the highest risk of being deleterious to human health. MPs, on the other hand, are typically detected only in the gastrointestinal tract of marine organisms, commonly not consumed. Small low trophic organisms, like mussels, shellfish, and mesopelagic species, however, are often eaten whole and could thus contribute to ingested MPs [30,34,35]. Despite these preliminary studies, the VKM concluded that the available information regarding MPs and NPs does not provide a sufficient basis to characterize potential toxicity in humans [32].

Acknowledging the lack of scientific certainty and the fragmentation of the preliminary results, the cited research reaches a consensus on the urgency of intervening at the regulatory level. It does so by applying the precautionary principle in the areas where there is still insufficient scientific evidence, especially regarding the impacts of NPs on food safety and human health, “given the unavoidable increase in the coming decades of micro and nanoplastics in the marine environment” [36].

A legal reform that seeks to effectively tackle the plastic emergency should take into consideration the highlighted advances in research, and by doing so contribute to the improvement of the science-policy dialogue in the subject matter.

### 2.3. Understanding the State-Of-The-Art of Plastics Regulation

Plastic governance—when compared to the governance of other environmental problems—has at least three shortcomings: 1. It is not yet a fully, integrated and globally steered process [10]; 2. It is mainly enacted through soft-law mechanisms; and 3. It spans a time range of 10 years (up until 2030), a circumstance that hinders prompt and decisive action. Despite the efforts to tackle marine litter at the international level [37], the initiatives undertaken so far appear to be characterized by very weak enforceability (they belong to the category of ‘soft law instruments’) while covering a very wide margin of time intervention. Similar drawbacks are also present at the EU level, where the EU Plastics Strategy has been launched under the umbrella of the Green Deal and the Circular Economy Plan [38]. Beyond the statement of general principles, only one among the announced actions has been implemented to date. It is the Directive EU 2019/904 on the reduction of the impact of certain plastic products on the environment [39]. Hence the concern that the EU’s efforts—albeit commendable—are not sufficiently timely nor pervasively binding [40]. Accordingly, this lack of effective regulatory mechanisms, at both international and regional levels, illustrates that plastic governance provides, in fact, just another area where law is (too) far behind.

Despite the general reference to the principles of circular economy, silo, uni-sectoral and individualistic approaches still prevail in the choice of regulatory tools. This preference for individualistic approaches, in turn, creates several small bubbles of circularity with no communication between them: the circular system of plastic, of food, of human health, and even of the Agenda 2030. The challenge is to connect them, contributing to the ‘porosity of the governance soil’, and create synergies between sectors. The limitations of a singular and reductionist regulatory perspective can be clearly seen as one of the contributory causes of poor legal enforcement. This understanding reinforces the argumentation on the importance to unwaveringly push towards systematic and integrated approaches when facing the challenges posed by the complexity of sustainability.

### 2.4. Combining the Two Understandings with the Proposal of an Integrated Framework

The preliminary results of the project SECURE establish a blueprint which rectifies the fragmentation, and reductionist and weakly enforceable approaches to the MT plastic system [21]. Under this interdisciplinary project, research is conducted on the legal framework for environmentally conscious harvesting practices of new low-trophic marine species, the composition of nutrients and contaminants in these, and their effects on the gastrointestinal microbiome and cardiometabolic health [21]. The research spans complex sustainability challenges, such as environmental protection, food safety and security, human health, and innovation [21]. Thus, the SECURE team has been developing pioneering research approaches across different systematic disciplines under conditions of scientific uncertainty [41], offering insights to complex problem solving including those related to ocean plastics.

**Figure 1:**
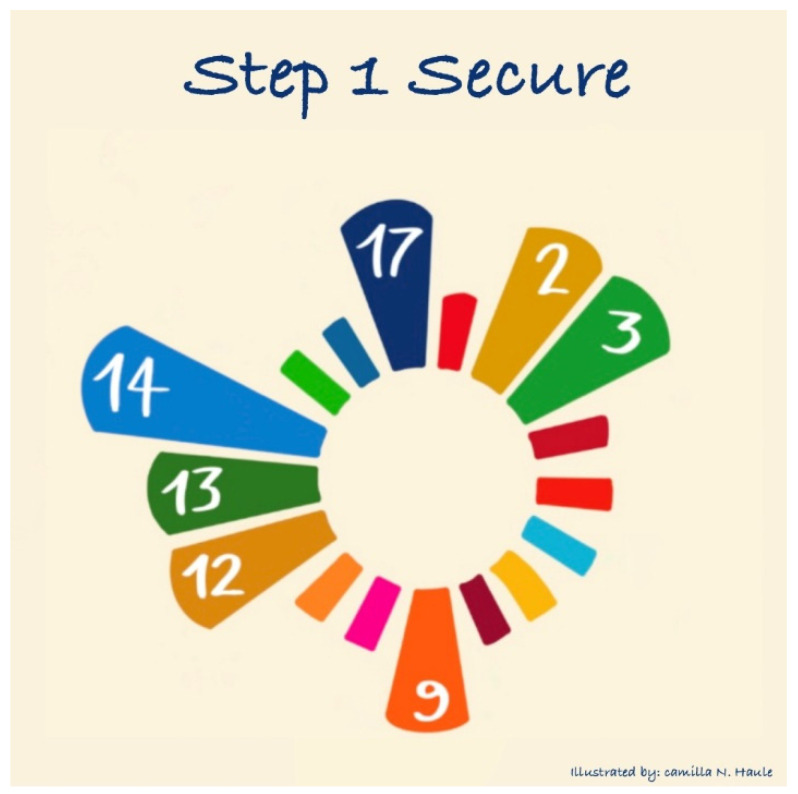
Illustration of Step 1 of the Project SECURE [41], representing the need to tackle novel marine resources through a systematic approach of the Agenda 2030 (with a main focus on SDG 14, on Life Below Water). Illustration for SECURE by Camilla Neema Haule 2021.

The first leg of the project focuses on the need to develop a matrix that conceptually encompasses the objectives of the Agenda 2030 and its 17 SDGs from a regulatory perspective on the one hand, and allows the upscaling of climate-smart practices applied to the ocean—as it is the case of the SECURE, on the other hand (Figure 1) [41]. The observed lack of a systematic mapping of the SDGs’ interactions and the dearth of climate-smart practices applied to the ocean spurred reflections on the need to create a critical conceptual framework as a reading key for the SDGs’ interactions and for upscaling successful climate-smart practices [41]. The application of a matrix based on systems thinking within a legal fabric opened the door to reflections about integrated regulatory approaches applied to the ocean.

**Figure 2:**
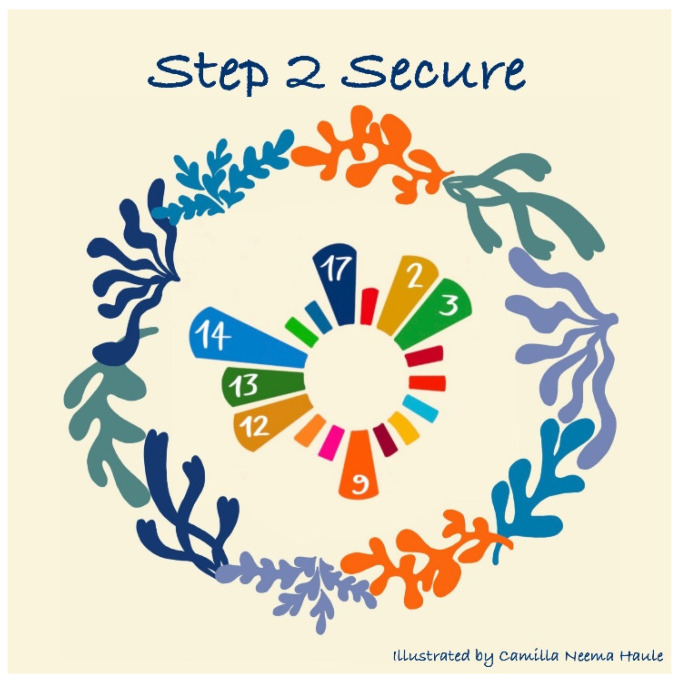
A case study relevant to the EU food on the integral ecology approach to food novelty applied to seaweed (frame of the illustration) through the lens of the systematic approach to the Agenda 2030 [42]. Illustration for SECURE by Camilla Neema Haule 2021.

Based on this, the second step of the legal research in SECURE has argued that the adoption of the specific systematic lens of integral ecology (IE) overcomes regulatory fragmentation caused by ‘glo*c*al’ environmental challenges (where the adjective ‘glo*c*al’ is meant to comprise both worldwide ranging and local issues) [36]. In particular, the integral ecology is the approach that observes the interconnections of the living systems, be they living organisms, social systems, or ecosystems with the aim to regulate them (Figure 2) [42]. Under IE, for example, there is no distinction between the natural world and human beings (both have agency, rights to life and regenerate), neither is there a distinction between health of the environment (water, plants, animals, soil) and health of humans. Through the lens of IE, a risk posed to the natural environment has consequences for all living beings [43]. In essence, IE advances the development and application of comprehensive and integrated approaches to sustainability issues, providing comprehensive, far-sighted, and flexible solutions for the environment—solutions that can restore the relationship, at multiple scales, with the earth [44].

**Figure 3:**
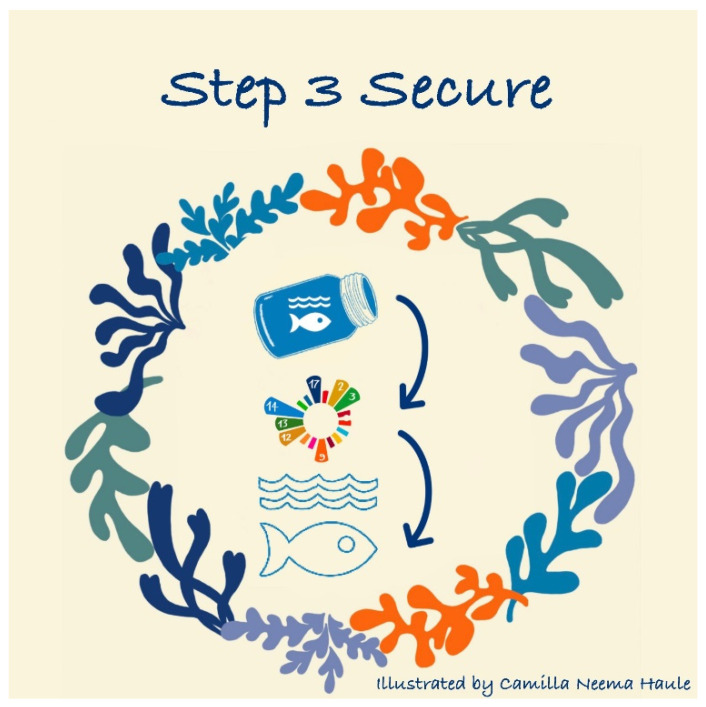
The systematic approach of the Agenda 2030 applied to ocean plastics: overcoming loopholes in regulation through a multilevel governance system applied to the SDG 14, on Life Below Water [41,42]. Illustration by Camilla Neema Haule for SECURE 2021.

The integral approach suggested by IE offers further insights into the complex problems and the uncertainties posed by plastics at sea, laying the foundation for systematic and integrated regulation. Construing the issue of plastic pollution through the lens of IE redefines the health of the ecosystem as the health of all beings, unlocking solutions in all those cases where the risks posed by plastics to food security and health are not yet proved beyond doubt. Grounded on the precautionary principle, a systematic and integrated regulation of plastic pollution at sea is justified by the need to prevent potential harms to the human rights to a healthy environment [43], healthy food, and to the rights of the Earth (and its living beings) to life, be respected and regenerate (Figure 3) [44].

## 3. Results

**Figure 4:**
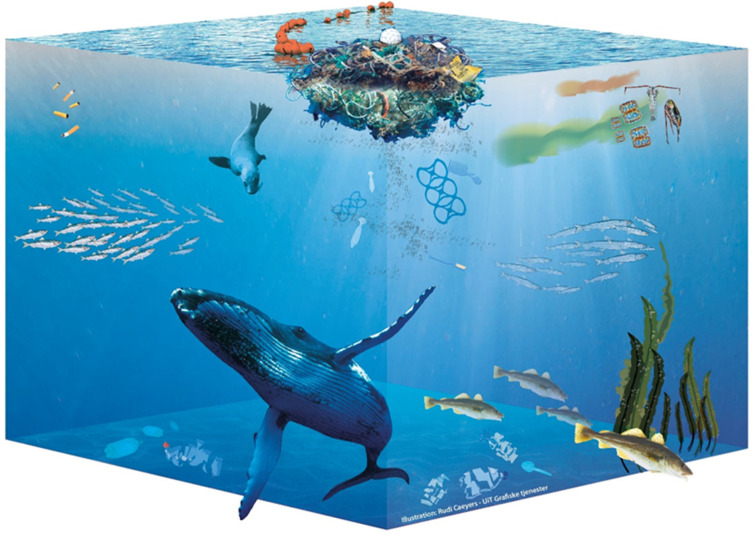
Plastic as a wicked problem, affecting the marine ecosystem as a whole. Illustration by Rudi Caeyers for SECURE, UiT Grafiske tjenester, 2021.

The suggestion for a systematic regulatory approach to ocean plastics stems from the research on the systemic approaches to environmental and societal challenges. The current narrative of understanding complex challenges is developed through a systemic approach based on resilient relationships rather than focusing on individual components of systems [45]. The issues related to sustainable development are often referred to by the scholars as ‘wicked problems’ (Figure 4) [46,47], defined as “trivial or lasting situations that cannot be overcome immediately due to their inner complexity or exogenous/endogenous relations” [46]. Plastic pollution, with its multiple potential impacts on the environment, food security, and food safety, as well as human health, is certainly one example of a wicked problem that needs to be tackled through a process-based, multi-tiered and systemic approach. The research, therefore, takes a crucial step forward towards the regulation of the wicked problem of plastics, concluding with recommendations for policymakers. Such recommendations are to be approved in the form of an MT governance framework.

A MT common regulatory framework tackling plastic pollution should:

- Contain a definition of plastics developed from the most advanced research in the field;
- Be grounded on the precautionary principle and the legitimacy of preventive actions, according to the international and European legal provisions [13];
- Be based on the premise that plastics pollution violates both human and nature rights;
- Provide the most detailed range of measures, with a clear indication of rights and duties (rights of lands, coasts, rivers, and marine ecosystem to be protected and duties of the human communities to act as nature’s guardians);
- Contain a wide range of bans, restrictions, and limits (e.g., broadening the current EU restrictions on certain single-use plastics to all single-use, and expanding such bans outside the EU), sanctions in case of infringement in coordination with the national criminal, civil, and administrative law systems;
- Promote, coordinate with, and implement systems that emphasize circular waste management practices and the use of alternative (renewable) resources as supplements or replacements for plastics; be coordinated with an effective reparatory justice system, allowing judicial and administrative reviews through actions initiated by the plurality of actors involved in the plastic justice movement; and
- Be constructed following the systems thinking approach and therefore develop multiple interconnections to the SDGs in the Agenda 2030 [47,48].

This research study demonstrates how a methodological approach applying systematic thinking to science and regulation helps to coordinate the most advanced results in science and the need to regulate the impacts of plastic pollution at sea. Multi-tiered and systemic methods for monitoring and ultimately restricting and banning plastics are key requirements to better understand, manage, and regulate such impacts, as well as to develop regulatory strategies and action plans at the international, regional, and national levels.

Studies on the complex challenges posed by sustainability highlight the movement from disciplinary regulatory baselines that tackle one objective at a time towards integrated approaches that acknowledge how the different problems in a system are interlinked [49]. Further research on empirical applications of the systemic approach is needed to assess the effectiveness of holistic approaches to tackle the complex problems of sustainability.

## 4. Discussion

### 4.1. Towards the Adoption of a Cross-Disciplinary Systems Thinking Approach

This study signifies a step forward in joint research concerning plastic pollution. Its reach extends to the exploration of the potential positive impacts of a systematic regulatory approach to plastics for the environment, human health, and in general the wellness of all living organisms. In this regard, it also marks a step forward in the project SECURE, at the crossroads of environment, food, and health. It does so by specifying in which terms systemic thinking applied to law and marine science can help tackle plastic pollution at sea.

Consequently, actors and disciplines can find a field of cross-cooperation and continue to develop ways to explore synergies. Two major reflections spur from this systemic approach: 1. How to effectively put it into practice; and 2. How to develop control mechanisms for its proper functioning.

The research community plays a key role in the solutions of the two questions. To test the robustness of systemic thinking and its applications to cross-boundaries issues, it is necessary to invest efforts towards systemic research. The steps of systemic research include developing standards to conduct systemic research, the establishment of a common language (to the extent possible) between the different research disciplines, as well as the implementation of review panels that overview, assist, and review the developments of the research. Examples of the latter can be found within research in healthcare [50].

Such review panels are to be formed by researchers, as well as key users and stakeholders, with the skills and content knowledge to produce and assess systematic research. The tasks in the systemic research fields should therefore be conducted and reviewed by multiple individuals with a wide range of expertise, from scoping studies to developing systemic methods that synthesize the research findings. To reinforce the role of systems thinking in solving complex problems, it would be desirable to introduce such an approach also in education, across the physical, natural, and social sciences [51,52].

While additional research is needed to build empirical support for effective approaches to systems thinking, this article has set the groundwork to facilitate the adoption of such an approach in research, education, and ultimately in decision-making.

Improving the use of systemic research and reviews in decision-making has the potential to provide multi-faceted interventions to limit the impacts of plastic pollution at sea. Collaboration between hard scientists and legal researchers is essential for developing consensus and clarity on regulatory strategies and action plans, thus maximizing the coordination and effectiveness of the intervention measures [9].

Finally, the advancements in systemic approaches are expected to further not only the plastic pollution research field, but also all the related and expectedly impacted ambits, such as food security, food safety, and human health.

### 4.2. From Legislation to Implementation: A Comparative Overview of the First Implementing Efforts of Directive EU 2019/904

France and Germany offer two virtuous examples of implementation of the Directive EU 2019/904 [39]. In particular, France has been a pioneer in the introduction of obligations on plastic waste, with the approval of the Law on the Circular economy (Law No. 2020-105 of 10 February 2020 — ‘Circular Economy Law’) [53]. Among the measures introduced by the law to curb plastics waste, it is worth listing the progressive ban from January 2021 on single-use plastic products; the ban on the import and manufacture of single-use plastic bags intended for sale or for giving-out for free; general limits on the use of plastic [53].

In the wake of implementing the European law, Germany has adopted the Ordinance on Single-Use Plastics (Einwegkunststoffverbotsverordnung, EWKVerbotsV), entered into force on 3 July 2021 [54]. The EWKVerbotsV implements Article 6 (1), (2) and (4) of Directive EU 2019/904, requiring Member States to ensure that the single-use plastic products listed in part C of the Annex to that Directive, whose closures and lids are made of plastic, are placed on the market only if the closures and lids remain attached to the containers during the period of use. The Ordinance also implements Article 7 (1) and (3) of Directive (EU) 2019/904, requiring the EU member states to ensure that the containers listed in Part D of the Annex are only placed on the market if they bear a marking on the packaging or on the product itself.

Both examples bode well in terms of improvements of a MT common regulatory framework, involving multi-actors and multi-action strategies.

## 5. Conclusions

The known and unknown challenges posed by plastics pollution are putting pressure on hard scientists and legislators. Bridging scientific advancements and regulatory needs is the challenge that researchers are asked to address in a subject that is still, defined “at its infancy” [10]. The complex challenges that plastics pollution poses require the adoption of systemic approaches that interpret and analyze the most advanced research from marine biology, food sciences, and law. Within such a scenario, methodologies based on systems thinking and integral approaches need to be adopted as the key to intervening, marking a significant and effective regulatory change in a way that can expectantly neutralize or at least limit the devastation of plastic pollution in the ocean. The model suggested in this article has the potential to be applied in the different fields of environmental protection, food security, food safety, and human health to organize information about complex scenarios holistically and effectively. This method transcends the plastic pollution scene: systemic approaches can be implemented by other scholars and researchers in different sectors, thereby facilitating cross-disciplinary discussions on systemic environmental and societal challenges. Systemic research dealing with cross-cutting issues, such as environmental, food, and health challenges requires coordinated efforts among a multitude of actors and institutions across sectors, levels, and jurisdictions. Achieving environmental sustainability, health, food security, and safety for all beings requires a whole-of-system approach and coordinating efforts from all the actors involved.

While additional empirical studies are needed to assess whether the implementation of the described model can effectively address the challenges posed by marine pollution, this study has set the baseline for further exploration of the systems thinking practices to solve wicked interconnected problems.

## Data Availability

Not applicable.
